# Nutritional Supplementation Inhibits the Increase in Serum Malondialdehyde in Patients with Wet Age-Related Macular Degeneration

**DOI:** 10.1155/2017/9548767

**Published:** 2017-01-24

**Authors:** Toshiyuki Matsuura, Kei Takayama, Hiroki Kaneko, Fuxiang Ye, Hiroshi Fukukita, Taichi Tsunekawa, Keiko Kataoka, Shiang-Jyi Hwang, Yosuke Nagasaka, Yasuki Ito, Hiroko Terasaki

**Affiliations:** ^1^Department of Ophthalmology, Nagoya University Graduate School of Medicine, Nagoya 466–8550, Japan; ^2^Department of Ophthalmology, Shanghai First People's Hospital, Shanghai Jiao Tong University School of Medicine, Shanghai 200240, China; ^3^Laboratory of Bell Research Center-Department of Obstetrics and Gynecology Collaborative Research, Nagoya University Graduate School of Medicine, Nagoya 466-8550, Japan

## Abstract

*Purpose.* To compare serum levels of malondialdehyde (MDA) in patients with wet age-related macular degeneration (wAMD), patients with dry AMD (dAMD), and patients without AMD and to evaluate the efficacy of nutritional supplementation for treating elevated serum MDA in patients with wAMD.* Methods.* MDA levels were measured in sera from 20 patients with wAMD, 20 with dAMD, and 24 without AMD. Patients with wAMD were randomized to receive or not receive nutritional supplementation (10 patients in each group), and MDA levels were measured after 3 months of treatment.* Results.* MDA levels in patients with wAMD were significantly greater compared with patients without AMD. In eyes with wAMD, there was a significant correlation between MDA levels and choroidal neovascularization lesion area. Serum MDA levels decreased in most patients that received supplementation and significantly increased in those who did not.* Conclusion.* Baseline serum MDA levels were elevated in patients with wAMD, and MDA levels were directly correlated with choroidal neovascularization lesion area. In addition, nutritional supplementation appeared to exert a protective effect against oxidative stress in patients with wAMD.

## 1. Introduction

Age-related macular degeneration (AMD) is a leading cause of blindness in developed countries [1, 2]. AMD is classified as either wet AMD (wAMD) or dry AMD (dAMD) according to its pathophysiology [3]. wAMD is characterized by choroidal neovascularization (CNV) and an increase in intraretinal/subretinal fluid which is strongly associated with the overexpression of vascular endothelial growth factor (VEGF). dAMD is characterized by the atrophy of retinal pigment epithelium (RPE) and its advanced form is called geographic atrophy (GA) [4, 5]. Multiple risk factors, including obesity [6], hypertension [7], smoking [8], and light exposure [9, 10], have been shown to contribute to the pathogenesis of AMD, presumably by inducing oxidative stress [11–13].

Nutritional supplements, that is, Ocuvite PreserVision with Lutein, have shown to provide therapeutic benefits in both wAMD and dAMD [14–16]. The Age-Related Eye Disease Study 2 (AREDS 2) found that high-dose zinc/antioxidant supplementation inhibited the progression of early stage AMD to late stage AMD compared with placebo [17, 18]. A previous study demonstrated that mean serum levels of cysteine, an oxidative stress marker, decreased in study participants on a regulated diet that received a 5-day course of antioxidant and zinc supplementation [19].

Malondialdehyde (MDA) is a highly reactive three-carbon dialdehyde product of polyunsaturated fatty acid (PUFA) peroxidation by free radicals [20, 21]. Consuming high levels of linoleic acid, a *ω*-6 fatty acid and the most abundant dietary PUFA, is a risk factor for multiple types of cancers [22] and heart diseases [23]. Photoreceptor outer segments are rich in unsaturated fatty acids. As MDA is generated from the oxidation of unsaturated fatty acids, MDA is present in drusen, the extracellular deposits that accumulate in AMD eyes [24, 25]. MDA is used as a biological marker of oxidative stress [26], suggesting that serum MDA levels are likely to be elevated in patients with AMD compared with healthy subjects [27–29]. Consistent with this hypothesis, MDA exerts cytotoxic effects and upregulates VEGF expression in RPE cells in vitro [30, 31]. We previously demonstrated that not only is MDA a marker of AMD but it also induced autophagy dysregulation and VEGF secretion in AMD eyes. Furthermore, higher levels of dietary linoleic acid intake promoted CNV progression in mice with high MDA levels [32]. In this study, we further analyzed the relationship of clinical characteristics and oxidative stress levels in patients with AMD. In addition, we investigated the efficacy of antioxidant nutritional supplements in reducing serum MDA levels.

## 2. Materials and Methods

### 2.1. Patients

We prepared serum samples from patients with wet AMD (wAMD group), patients with dry AMD (dAMD group), and individuals without AMD (control group). All of the study patients were >50 years old and their axial length was >23.0 mm and <26.0 mm. Patients with polypoidal choroidal vasculopathy, retinal angiomatous proliferation, maculopathy with myopic CNV, or CNV based on angioid streaks or patients with axial length >26.0 mm or <23.0 mm were excluded from the study. The diagnosis of wAMD and dAMD was established on the basis of age (over 50 years), clinical examination, fundus photography, optical coherence tomography, and fluorescein fundus angiography as previously described [33, 34]. Patients with wAMD in one eye and dAMD in the other eye were excluded from the study. Control sera were obtained from patients with other ocular diseases, including cataract, glaucoma, retinal detachment, macular hole, and epiretinal membrane. After baseline measurements were obtained, patients in the wAMD group were divided into two groups; patients in the wAMD group were free to choose whether nutritional supplements should be taken. Patients who took the nutritional supplement were referred to as S (+) and those who did not take it were referred to as S (−). The nutritional supplement (Ocuvite PreserVision with Lutein, Bausch & Lomb, Rochester, New York City, The United States of America), which was commercially available at the time of the study, contains vitamin C (408 mg), vitamin E (241 mg), zinc (30 mg), and lutein (9 mg). The participants received the supplement once daily for 3 months. The study was approved by the Nagoya University Hospital Ethics Review Board (#2012-0340-3), and written informed consent was obtained from each patient prior to obtaining the first serum samples.

### 2.2. Best-Corrected Visual Acuity

The best-corrected visual acuity (BCVA) was measured using a standard Japanese visual acuity chart. The decimal BCVA was converted into the logarithm of the minimum angle of resolution (logMAR) for the statistical analysis.

### 2.3. Fluorescein Angiography Imaging and Evaluation of Choroidal Neovascular Lesions

Fluorescein angiography (FA) was recorded for all patients in the wAMD group using cSLO (Heidelberg Retina Angiograph, HRA 2, Heidelberg Engineering, Dossenheim, Germany) as previously described [35–37]. To evaluate the area of the CNV lesion, we traced the border of the area of hyperfluorescein in images captured at 5 min and quantified the pixels using NAVIS bundled software (Nidek Co. Ltd., Aichi, Japan). The measurements were conducted by two observers (Toshiyuki Matsuura and Kei Takayama) and both observers were blinded to the patients' clinical features. The area of one pixel in the FA images was defined as 0.0004 mm^2^, and the measurements were converted from pixels to the area (mm^2^).

### 2.4. Fundus Autofluorescence Imaging and Evaluation of Geographic Atrophy (GA)

Fundus autofluorescence (FAF) was recorded using a cSLO (Heidelberg Retina Angiograph, HRA 2, Heidelberg Engineering, Dossenheim, Germany) as previously described [38, 39]. An optically pumped solid-state laser was used to generate the excitation, and emitted light with a wavelength >500 nm was detected using a barrier filter. The images were immediately digitized. Then, they were processed using a flexible frame processor and displayed on a computer screen. The FAF images were recorded in accordance with a standard operating procedure. The red-free reflection mode was used for focusing, and a series of 30 × 30° images were acquired. To evaluate the area of GA from the FAF images, we traced the border of the dark area in the image and measured the pixels using NAVIS bundled software (Nidek Co. Ltd., Aichi, Japan). The measurements were conducted by two observers (Toshiyuki Matsuura and Kei Takayama) who were blinded to the patients' clinical features. The area of one pixel in the FAF images was 0.0004 mm^2^, and the measurements were converted from pixels to the area (mm^2^).

### 2.5. MDA Levels

MDA levels in patient sera were measured at baseline. Then, patients in the wAMD group were randomized to the S (+) group or S (−) group, and sera that were obtained were measured 3 months later. Serum levels of MDA were measured using an OxiSelect MDA Adduct ELISA Kit (STA-332; Cell Biolabs, San Diego, CA, USA) as previously described [32, 40]. Duplicate evaluations were performed for each sample.

### 2.6. Statistical Analysis

All of the data are presented as the mean ± the standard error of the mean. The Mann–Whitney *U* test was used to compare data between the wAMD, dAMD, and control groups and to compare MDA levels at baseline and after 3 months. Spearman's correlation was used to detect the correlation between MDA levels and age, logMAR BCVA, CNV area, and GA area. *P* < 0.05 was considered statistically significant.

## 3. Results

### 3.1. Patient Characteristics

The study included 20 patients in the wAMD group (10 males, mean age 71.8 ± 11.0 years), 20 patients in the dAMD group (13 males, mean age 70.6 ± 12.5 years), and 24 patients in the control group (11 males, mean age 68.2 ± 10.2 years). The patient characteristics were presented in Table 1. The mean logMAR BCVA was 0.29 ± 0.21, 0.24 ± 0.24, and 0.22 ± 0.29 in the wAMD, dAMD, and control groups, respectively. Mean levels of MDA were 9.94 ± 1.53 pmol/mL, 9.30 ± 0.92 pmol/mL, and 9.04 ± 0.96 pmol/mL in the wAMD, dAMD, and control groups, respectively. Mean CNV area in the wAMD group was 4.65 ± 3.66 mm^2^, and mean GA area in the dAMD group was 2.29 ± 1.80 mm^2^. The patient characteristics of the three groups are presented in Table 2. Individual value of MDA-protein adducts and patient information of S (+) and S (−) group are presented in Table 3. The mean logMAR BCVA was 0.30 ± 0.25 and 0.28 ± 0.17 in the S (+) and S (−) groups, respectively. Mean levels of MDA were 10.34 ± 2.03 pmol/mL in the S (+) group and 9.54 ± 0.70 pmol/mL in the S (−) group. The mean area of CNV lesions was 4.70 ± 4.09 mm^2^ in the S (+) group and 4.62 ± 3.41 mm^2^ in the S (−) group. There were no significant differences between the S (+) and S (−) groups in any of the parameters evaluated.

### 3.2. MDA Level

**Figure 1:**
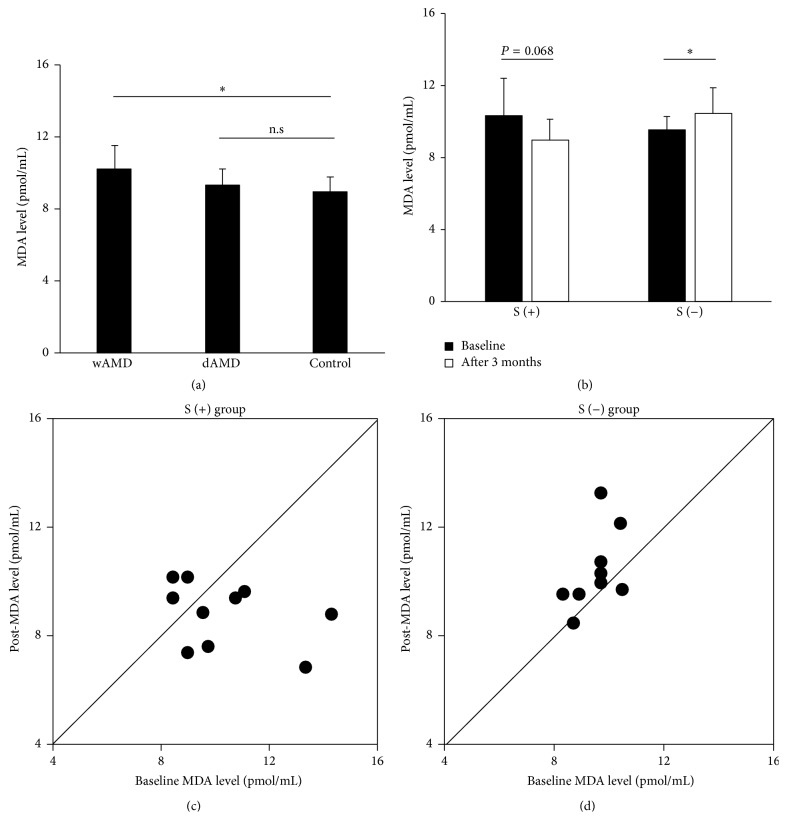
Serum MDA levels in the study population and changes in MDA levels in wAMD patients that received nutritional supplementation. (a) Higher serum MDA levels were observed in the wAMD group. (b) MDA levels tended to decrease in wAMD patients that received supplementation for 3 months. (c) MDA levels decreased in 7 of the 10 patients in the S (+) group and increased in the remaining 3 patients. (d) MDA levels decreased in 2 of the 10 patients in the S (−) and increased in the other 8 patients. ^*∗*^*P* < 0.05.

To investigate the relationship between MDA levels and AMD, we evaluated serum MDA levels at baseline and after 3 months of treatment (Figure 1). We observed a 15.0% increase in mean serum MDA in the wAMD group (9.94 ± 1.53 pmol/mL) compared with the control group (9.04 ± 0.96 pmol/mL, *P* = 0.031). An increase in MDA was also observed in the dAMD group (9.30 ± 0.92 pmol/mL) compared with the control group, although the difference was not significant (*P* = 0.12; Figure 1(a)).

Mean levels of MDA in the S (+) group decreased from 10.34 ± 2.03 pmol/mL at baseline to 8.88 ± 1.18 pmol/mL after 3 months of supplementation (*P* = 0.064). Mean levels of MDA in the S (−) group significantly increased from 9.54 ± 0.70 pmol/mL at baseline to 10.41 ± 1.36 pmol/mL after 3 months (*P* = 0.012) (Figure 1(b)). MDA levels decreased in 7 of the 10 patients in the S (+) group and increased in the other 3 patients (Figure 1(c)). In contrast, MDA levels decreased in 2 of the 10 patients in the S (−) group and increased in 8 patients (Figure 1(d)).

### 3.3. Correlation between Serum MDA Levels and Patient Characteristics

**Figure 2:**
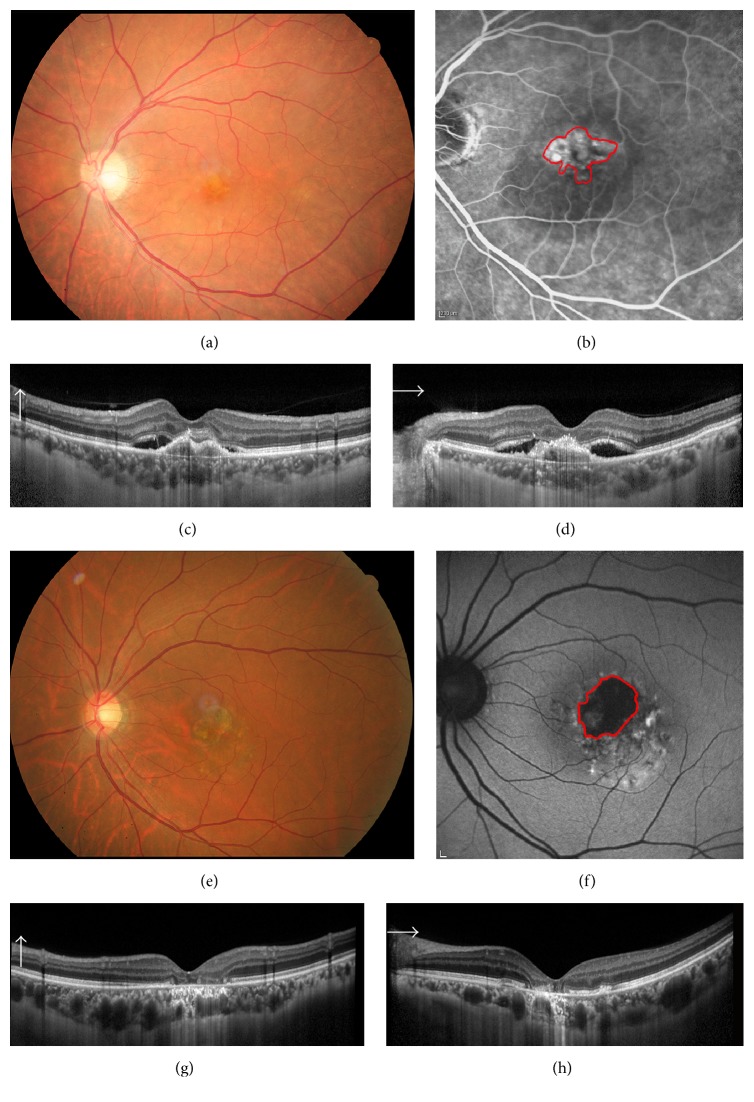
Representative images of wet AMD and dry AMD. (a, b) The color (a) and fluorescein angiography (FA, b) images of a representative case of wet AMD. The CNV lesion area in FA images was measured. (c, d) Representative case of dry AMD. Geographic atrophy area in the autofluorescein angiography image (d) was measured. (e, f) images are the OCT images of wet AMD and (g, h) are of dry AMD.

Next, we examined the correlation between baseline MDA levels and age, logMAR BCVA, CNV area, and GA area. Representative images of CNV in eyes with wAMD and GA area in eyes with dAMD are shown in Figure 2. In the representative eye with wAMD, logMAR BCVA was 0.30, baseline serum MDA was 10.28 pmol/mL, and CNV area was 3.27 mm^2^ (Figures 2(a) and 2(b)). In the representative eye with dAMD, logMAR BCVA was 0.097, serum MDA level was 8.90 pmol/mL, and CNV area was 3.40 mm^2^ (Figures 2(c) and 2(d)).

**Figure 3:**
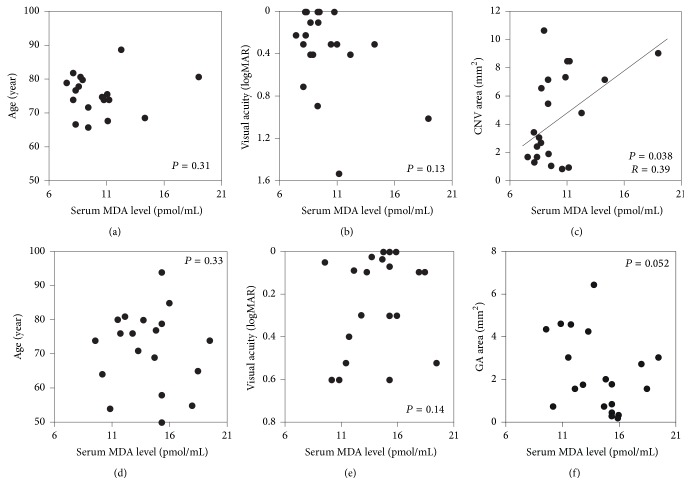
Correlation between MDA levels and patient characteristics in the wet AMD and dry AMD groups. (a–c) In the wet AMD group, serum MDA levels and choroidal neovascularization (CNV) lesion area were significantly correlated, but MDA levels were not correlated with ages or visual acuity. (d–f) In the dry AMD group, there was no significant correlation between serum MDA levels and age, visual acuity, or geographic atrophy (GA) area.

In the wAMD group, there was a significant correlation between baseline MDA levels and CNV area (*P* = 0.038, *r* = 0.39, Figure 3(c)), although we did not detect significant correlations between MDA levels and age (*P* = 0.31, Figure 3(a)) or logMAR BCVA (*P* = 0.13, Figure 3(b)). In the dAMD group, baseline MDA levels were not significantly correlated with age, logMAR BCVA, or GA area (*P* = 0.33, 0.14, and 0.052, resp.) (Figures 3(d)–3(f)).

## 4. Discussion

The association between AMD and oxidative stress both in vitro and in vivo has been demonstrated in several studies [12, 41, 42]. We previously demonstrated that MDA is not only a marker of AMD but also a direct contributor to the pathogenesis of AMD [32]. Prospective studies demonstrated that serum markers of oxidative stress are low in patients taking various nutritional supplements, including antioxidants such as lutein, vitamin C, vitamin E, *β*-carotene, and zinc oxide. However, only one study evaluated a 5-day course of nutritional supplementation and included a relatively small number of patients [19]. In the present study, we measured MDA levels in the sera using ELISA and regarded them as a systemic oxidative stress marker because a previous report describing the complements binds MDA accumulating in the drusen of the eyes with AMD and complement factor H genetically plays an important role in this complement-MDA cleavage [25]. We were able to demonstrate the usefulness of supplementation by measuring MDA-protein adducts in this study. However, free MDA-protein levels measured by high performance liquid chromatography or 4-hydroxy-2-nonenal are considered as more reliable biomarkers of lipid peroxidation in the human plasma [26, 43]. More reliable correlations could be estimated by measuring them. In the present study, we demonstrated that mean serum MDA levels decreased in patients on a regulated diet that received nutritional supplementation, Ocuvite PreserVision with Lutein, for three months, whereas mean levels increased in patients on a regulated diet with no nutritional supplementation. As systemic oxidative stress is a key contributor to the pathogenesis of AMD, these findings suggest that nutritional supplementation might have inhibited systemic oxidative stress in these patients.

In patients with wAMD, MDA levels were significantly correlated with CNV area (Figure 3(c)). CNV is defined as neovascularization resulting from oxidative stress-induced damage to the choroid and RPE [44–46]. We previously demonstrated that MDA levels in the RPE and choroid of AMD patients were significantly elevated compared with control subjects and that MDA administration induced the upregulation of VEGF expression in RPE cells and induced an increase in CNV volume in vivo [32], suggesting that serum MDA levels are correlated with CNV area. To measure CNV lesion area, FA/ICGA were performed in the wAMD group, as previously described. Changes in the CNV area in the S (+) and S (−) groups would be detectable by FA/ICGA after 3-month observation, although FA/ICGA was not performed in the present study. From OCT images, the CNV area was considered to show no remarkable change after 3-month observation. Therefore, the relation between CNV area expansion and MDA change could not be compared. We did not detect a correlation between MDA and GA area in the dAMD group (Figure 3(f)). GA is defined as a loss in RPE area [1, 47]. The RPE maintains the health of the retina by providing protection from oxidation [48, 49]. GA can take more than 6 years to develop, and it arises as a consequence of long-tern exposure to stress [33], rather than current levels of stress. These findings are consistent with the lack of a correlation between GA area and serum levels of MDA in this study. Although we measured only MDA-protein adducts in this study, measuring one more biomarker, for example, 4-HNE, is preferable in the further study.

There are several limitations of the study worth noting. First, the present study included small number of patients and the observational period was only 3 months. More patients and long-term studies are needed to confirm our findings. In addition, the control group should also be treated by the same nutritional supplementation to have a proper control. A better method would be that the patients in the control groups take same supplementation after 3 months and it should be confirmed that their MDA levels will be also reduced after taking supplementation. The present study showed only “tendency (*P* = 0.068),” not “significant difference (*P* < 0.05),” of supplementation for reducing MDA levels after 3 months. It is possible that a longer observation period may result in “significant difference” in MDA levels between S (+) and S (−) groups. However, other factors, that is, food or stress, could affect systemic oxidative stress during the long-time observation. Second, it was not elucidated whether nutritional supplementation directly reduces MDA level in the eye. We previously reported that high levels of MDA were observed in human RPE/choroid obtained from donor eyes from several Eye Banks. The AREDS demonstrated the efficacy of supplementation in the prevention of AMD progression over a period of 5 years [17, 50]. It is difficult to prospectively study the effect of nutritional supplementation on MDA levels in human eyes.

In conclusion, we demonstrated that elevated serum MDA levels were directly associated with the area of CNV lesions in eyes with wAMD and that nutritional supplements appear to protect the eyes from systemic oxidative damage. MDA might be a valuable marker of oxidative stress. The present study is a retrospective short-observation study including small number of patients. To obtain more reliable value of supplementation for AMD and systemic oxidative stress, a double-blinded prospective randomized study of supplementation is necessary.

## Figures and Tables

**Table 1:** Patient characteristics in wAMD, dAMD, and control groups.

	wAMD	dAMD	Control
Number	20	20	24
Male/female	10/10	13/7	11/13
Mean age (years)	71.8 ± 11.0	70.6 ± 12.5	68.2 ± 10.2
Mean logMAR BCVA	0.29 ± 0.21	0.24 ± 0.24	0.22 ± 0.29
Mean MDA level (pmol/mL)	9.94 ± 1.53	9.30 ± 0.92	9.04 ± 0.96
Mean CNV area (mm^2^)	4.65 ± 3.66		
Mean GA area (mm^2^)		2.29 ± 1.80	

**Table 2:** Patient characteristic in the S (+) and S (−) groups.

	S (+)	S (−)
Number	10	10
Male/female	5/5	5/5
Mean age (years)	72.0 ± 9.7	71.6 ± 10.1
Mean logMAR BCVA	0.30 ± 0.25	0.28 ± 0.17
Mean MDA level (pmol/mL)	10.34 ± 2.03	9.54 ± 0.70
Mean CNV area (mm^2^)	4.70 ± 4.09	4.62 ± 3.41

**Table 3:** Patients of S (+) and S (−) group.

#	Age (years)	Sex	logMAR BCVA	CNV area (mm^2^)	Before MDA (pmol/mL)	After MDA (pmol/mL)
S(+) group						
1	81	M	0.70	9.09	8.94	7.41
2	81	F	0.70	6.70	8.45	10.23
3	80	M	0.30	1.51	8.45	9.46
4	74	M	0.00	11.41	10.74	9.46
5	74	M	0.10	7.37	9.71	7.68
6	73	F	0.40	1.45	9.52	8.90
7	72	F	0.22	0.85	11.07	9.46
8	69	M	0.30	7.27	14.28	8.86
9	68	F	0.30	1.01	8.94	10.23
10	50	F	0.00	0.30	13.29	6.91

S(−) group						
1	94	F	0.30	0.85	9.71	9.98
2	81	F	0.30	6.61	8.69	8.51
3	80	M	0.40	10.69	8.90	9.54
4	78	M	0.10	1.73	9.71	10.41
5	74	M	0.22	5.68	8.32	9.54
6	74	F	0.70	5.53	9.71	10.74
7	67	F	0.10	1.25	10.41	12.17
8	59	F	0.22	8.53	9.71	13.30
9	55	M	0.22	1.12	9.71	10.23
10	54	M	0.22	4.18	10.48	9.72
